# Perceptions of Cancer Through the Ages—From Hippocratic Oncology to Precision Cancer Medicine

**DOI:** 10.3390/biom14111383

**Published:** 2024-10-30

**Authors:** Spyros Retsas

**Affiliations:** Formerly Westminster Hospital, Chelsea and Westminster Hospital and Charing Cross Hospital, London SW10 9NH, UK; eupalineios@gmail.com

**Keywords:** cancer, oncology, antiquity, precision, medicine

## Abstract

The unravelling of the human genome created new perceptions of the origin and evolution of diseases, and for cancer in particular, it established the notion that neoplasia has been a companion of life since its appearance on Earth. It is not surprising that neoplasms, in various forms, develop in numerous species of animals and even in plants. Unmistakable accounts of cancer with clinical features as are understood today begin in the 5th c. B.C. The principles and practice of the Hippocratic and Galenic tradition dominated cancer care virtually into the 20th century. Advanced sequencing technologies at the dawn of the 21st century generated new therapeutic opportunities with immunotherapy, oncolytic virotherapy, and gene transfer, with the latter especially being used in cases of hereditary cancer.

## 1. Introduction

The unravelling of the human genome [1] created new perceptions of the origin and evolution of diseases, and for cancer in particular, it established the notion that neoplasia has been a companion of life since its appearance on Earth.

It is not surprising that neoplasms, in various forms, develop in numerous species of animals [2,3] and even in plants, with the latter, when afflicted by phytoviruses or other infections, developing “*unnatural growths*”, as Galen, the 2nd-century A.D. physician, would have articulated.

The ΤOR (target of rapamycin) signalling pathway is ubiquitous in eukaryotic cells and has been evolutionarily conserved in fungi, plants, animals, and humans [4,5]. It is obvious that dysregulations of these functions by exogenous or genetic causes can cause, among other things, uncontrolled cell proliferation, culminating in neoplasia.

The question, therefore, is not whether cancer existed in ancient societies—(it did!)—but whether the physicians of antiquity recognised it and described it as a distinct nosologic entity. There are inferences to growths in Egyptian papyri, variously interpreted as cancer, but the evidence is conjectural [6,7]. In Chinese language, according to Shan Jiang, of the Institute of Medical Humanities, Peking University, Beijing, China and Visiting Fellow at the Institute of Advanced Studies, University College, London (UCL), the *terms 肿瘤zhongliu* and *瘤liu* have broad meanings, encompassing all types of tumours, benign and malignant. On the other hand, *ai* narrowly refers to malignant neoplasia [8]. Shan Jiang’s report relates to data from the Chinese classical era (around 25–220 CE), specifically based on texts in *The Yellow Emperor’s Inner Canon*.

According to K.M.N. Kunzru and Radha Bhat, there is no actual concept of “neoplasm” in Ayurveda, the ancient Indian system of medicine. The surgeon–physician, *Suśruta*, however, describes several tumour/neoplasm-like lesions in clinical and therapeutic detail in his monumental compendium, *Suśrutasamhitã*. *Suśruta* trained, practised, and taught in the North Indian city Kãśi, modern Varanasi, sometime in the mid-1st Millennium BCE [9].

Unmistakable accounts of cancer with clinical features as are understood today begin in the 5th c. B.C. with Hippocrates of Cos and evolved into the 7th c. A.D. with Paul of Aegina. At least fifteen physicians of this period describe the incidence of cancer, predominantly in advanced age, the clinical characteristics, its life-threatening course, and attempts to treat it [10].

The first thorough and systematic study of the ancient Greek literature on cancer is credited to the distinguished historian of medicine, Aristotle P. Kouzis, who, in 1902, as an Associate Professor of the History of Medicine at the National University of Athens, published a monograph entitled “CANCER AND THE ANCIENT GREEK PHYSICIANS” (*O ΚAΡΚΙΝOΣ ΠAΡA ΤOΙΣ AΡΧAΙOΙΣ ΕΛΛHΣΙΝ ΙAΤΡOΙΣ*) [11]. Aristotle Kouzis became, later in life, President of the Academy of Athens.

In the Hippocratic Corpus, we find the terms *Καρκίνος* (cancer), *Καρκινοῦσθαι* and *Καρκινωθῆναι* (becoming cancerous) *Καρκίνωμα* (carcinoma), and *Καρκίνια* (small cancers), with a clear reference to the disease as we know it today [10].

The term *ὄγκος*, “tumour”, is mentioned in at least 12 passages in the literature attributed to Hippocrates, but without an obvious association with the disease *cancer.*

The physician Bacchius from Tanagra in the province of Boeotia, Greece, who wrote in the 3rd century B.C., included among the Hippocratic treatises one with the title “*On Carcinosis*” [10].

We may speculate that the author of this lost work would have discussed in detail the characteristics of cancer, and perhaps even the etymology of the term, which, however, we find much later in Galen.

In the existing works of the Hippocratic collection, we find the description of a characteristic clinical picture of breast cancer: “*The woman from Abdera developed breast cancer, and a blood-stained fluid was flowing from the nipple. When the discharge ceased, she died*”.

We have a clear reference to the frequency of cancer in advanced age and the fatal outcome of the disease. “*In the elderly such tubercles do not develop. But deep and superficial cancers appear, from which they die*” [10].

We encounter scepticism about the effectiveness of the treatment of deep-seated cancers: “*These who develop deep cancers are better left untreated. Because with treatment they perish quickly, whereas without treatment they live longer*”.

Reference is also made to the application of thermal energy, as in our day, for the control of cancerous tumours: “*He, with the carcinoma in the pharynx, was cured by us with cauterization*” [10].

And we encounter an observation, really admirable for the clinical acumen of the physicians of the Hippocratic era, which is often seen today in cases of immunosuppression, and which on occasion accompanies the development of neoplasia: “*Of all the ulcers that spread, herpetes are the least harmful, but they are difficult to eradicate in the presence of deep-seated cancers*” [10].

Some six centuries after Hippocrates, his commentator and admirer, Galen of Pergamum (120–200 A.D.?), provides a detailed clinical picture of cancer and the irrefutable testimony that the ancients knew about and understood the disease, which today is diagnosed clinically, not infrequently, before the histological documentation of a carcinoma [10].

Galen informs us about the nomenclature of diseases and characteristically writes the following:

“*Often a disease is named after the ailing part of the body, such as pleurisy, arthritis… sometimes from the symptom such as ileus, tenesmus, or spasm… and sometimes from both, such as headache or otalgia… and sometimes when it resembles something outside the body such as elephantiasis or “cancer” or polyp…*”

[10]

In another passage, he provides the etymology and the definition of cancer: “Carcinoma is a malignant and indurated tumour, ulcerated or non-ulcerated. It is named after the animal cancer” [10].

Galen elaborates further on the clinical picture of breast cancer, arguing that the distended veins that in some cases surround the tumour simulate in appearance the animal “*cancer* (crab): “… He adds, “…*and on the breasts we often saw tumours that looked exactly like the animal cancer. And just as the legs of the animal are lined up on either side of the body, so in this disease the distended veins of the unnatural growth form something resembling the crab (cancer)*” [10].

Galen knows that cancer develops in all organs; he recognises the high incidence of breast cancer during menopause, but he believes that as long as menstruation remains normal, women are not afflicted by the neoplasm [10].

Elsewhere, he repeats that carcinomas appear in all other parts of the body: “*Cancers develop mainly in the breasts but also in the genital organs of men and women. But cancer can appear in any part of the body, occult, ulcerated, malignant*.” [10].

It would appear, therefore, that the common anatomical cancer sites observed today were also common in antiquity.

Another term for cancer used by ancient Greek physicians is *Θηρίωμα*, “therioma”.

*Therion* in Greek means *wild beast*, and the word is probably used with an inference to the wild and uncontrolled nature of the disease or the monstrous appearance of some tumours [10].

Regarding the treatment of cancer, Galen boasts, “*…the early cancer we have cured, but the one that arose to considerable size, without surgery, no one has cured*”! [10]. The position of comma after *surgery* is crucial, but it is uncertain if this was the case in the original text.

This dogma of convenience dominates the therapeutic philosophy of cancer even in our day!

Elsewhere, Galen advocates the complete excision of the tumour approaching normal tissues: “*The aim of every surgical intervention is the complete excision of the abnormal growth, in a circle, approaching the margins of normal tissue*” [10].

The Alexandrian physician, Leonidas or Leonides (2nd century A.D.), a contemporary of Galen, gives a detailed description of a mastectomy for cancer, followed by instructions for post-operative care. “*… And in the case of carcinomas that do not adhere to the thorax,* he writes, *I operate in this manner. With the patient lying supine, I divide the healthy part of the breast above the carcinoma…and I cauterize the divided parts until haemostasis is achieved. Then, I incise the breast again, in depth, cauterizing the margins repeatedly until the bleeding is controlled…at the end and after the complete excision, I bring together the cauterised margins for the final reconstruction…*” [10,11].

## 2. What Did the Ancients Know About Metastasis?

The word *μετάστασις* is found for the first time in the work of the lyric poet, Simonides (6th–5th century B.C.). He laments the rapid and unexpected transition (*μετάστασις*) of man’s good fortune. Since then, the term has been used by philosophers, physicians, dramatists of comedy and tragedy, theologians, orators, and others, but with meanings very different from the current use of the word to mean *cancer dissemination*.

For example, Plato and Aristotle use metastasis when commenting on the transition, peaceful or violent, of a political system [12].

The word was introduced into the English literature for the first time towards the end of the 16th century, but in a rhetorical sense, not as a medical term [12,13].

In the Hippocratic Corpus, metastasis is mentioned at least nine times in several treatises, but without an apparent association with cancer!

For example, in the work ON THE SACRED DISEASE, one of the most important in the Hippocratic literature, the term metastasis is used in the discussion of pre-adolescent epilepsy (petit mal), which may cease during adulthood. In this case, metastasis also means the resolution of a disease or transition to health, rather than dissemination, as Galen explains in his comment on the relevant Hippocratic aphorism [12,13].

Although physicians had appreciated since the Hippocratic era that breast and uterine cancer could involve regional lymph nodes, the word used for this development was “sympathy” (*συμπάθεια*), not metastasis [11,12].

The establishment of the term metastasis, in the context of cancer dissemination, must have been significantly influenced by Ashworth’s publication in 1869, in which cancer cells in the bloodstream were mentioned for the first time [14]. Ashworth did not use the term metastasis but hinted at it by emphasising that “*cells observed in the blood invariably with those of cancer tend to illuminate the mode of origin of multiple tumours coexisting in the same individual*” [14].

The first time that metastasis was indisputably used to mean cancer dissemination was, in all probability, in 1911; Homer Gage, in his article on colon cancer, which was published in the *Boston Medical and Surgical Journal*, the precursor of the *New England Journal of Medicine*, wrote the following:

“*It has been frequently observed that cancer of the sigmoid seems to retain its local character much longer than cancer in other parts of the large intestine and that lymphatic involvement and visceral **metastases** occur much later*” [15].

## 3. Cancer Therapeutics in Our Era

The principles and practice of the Hippocratic and Galenic tradition dominated cancer care virtually into the 20th century with the precept “*catch it early and cure it*”, still an appealing concept repeatedly propagated in the medical literature, the press, and the media [16].

The dawn of the 20th century witnessed the emergence of a new treatment modality, radiotherapy, following the discovery of the X-rays by Wilhelm Conrad Röntgen in 1895 and of radium by Marie Curie in 1898 [17]. Radiotherapy developed in Europe, with the three major centres that pioneered and developed this new treatment modality located in Manchester in the UK, Villejuif (Institute Gustave Roussy) in France, and the Radiumhemmet in Stockholm [17]. Early in the 20th century, during the reign of King Gustav V, in Sweden, patients with cancer were entitled to free travel by train for treatment and follow up at the Radiumhemmet. This far-sighted approach, paradigmatic of Sweden’s leadership in social cohesion, was not only a humane treatment for cancer sufferers but allowed, at the same time, exceptional possibilities for the documentation of treatment outcomes, both positive and negative, of radiation therapy [17]. These were, presumably, the origins of Sweden’s uniquely reliable cancer registry.

In the multidisciplinary approach to cancer, radiation therapy, especially with the latest developments in stereotactic radiotherapy, often referred to as radiosurgery [17], and recent advances in particle therapy remain important components of cancer treatment for a significant number of selected patients with this disease.

It took two world wars in the last century and the emergence of the weapon of chemical warfare for oncology to enter the era of systemic treatment for a widely disseminated cancer. Mechlorethamine hydrochloride, also known as nitrogen mustard or mustine, targeting the cell’s DNA, in combination with vincristine, procarbazine, and prednisone, launched the new approach towards the conquest of widespread neoplasia, ultimately rendering Hodgkin’s lymphoma curable for the majority of patients [18]. Thomas Hodgkin (1798–1866), a pathologist at Guy’s Hospital in London, described the disease that bears his name in 1832, an incurable cancer in those days.

The introduction, in parallel, of the antimetabolite methotrexate, a folate antagonist, transformed a previously invariably lethal neoplasm, choriocarcinoma, into a treatable and eventually a highly curable cancer [19]. Anti-cancer drugs, derived initially from plants and subsequently produced semi-synthetically, include podophyllotoxin (etoposide) and the vinca alkaloids, vincristine, vinblastine, vindesine, and vinorelbine, which interact with tubulin and prevent microtubule formation, an essential component of cellular division.

A screening programme for phytopharmaceuticals culminated in the development of paclitaxel and docetaxel, two of the most effective drugs against refractory cancers. Synthetic analogues of the original drugs significantly reduced the cost of production and the impact on the environment. Additional examples of anti-cancer drugs derived from plants are harringtonine, homoharringtonine, and isoharringtonine, alkaloids from *Cephalotaxus harringtonia*; they exert antitumour activity by interfering with protein synthesis [20].

The inhibitory activity of cisplatin on cell division was noted in 1965 by the biophysicist Barnett Rosenberg [21]. Among other actions, it arrests DNA replication, leading to apoptosis or cell necrosis. Cisplatin entered clinical practice in 1978 and exhibited activity in a broad spectrum of neoplasms; it proved particularly effective in testicular carcinoma, another example of a curable neoplasm for the majority of patients with widespread disease.

Cisplatin’s efficacy in a broad spectrum of malignancies is limited by its nephrotoxicity and neurotoxicity, especially ototoxicity. It is also a highly emetogenic drug; this significant toxicity, which may have forced some patients to abandon an otherwise effective treatment, led to the development of new effective antiemetic agents that made treatment more tolerable [22]. The toxicity of cisplatin also led to the development of the synthetic analogues carboplatin and oxaliplatin [21].

**Figure 1:**
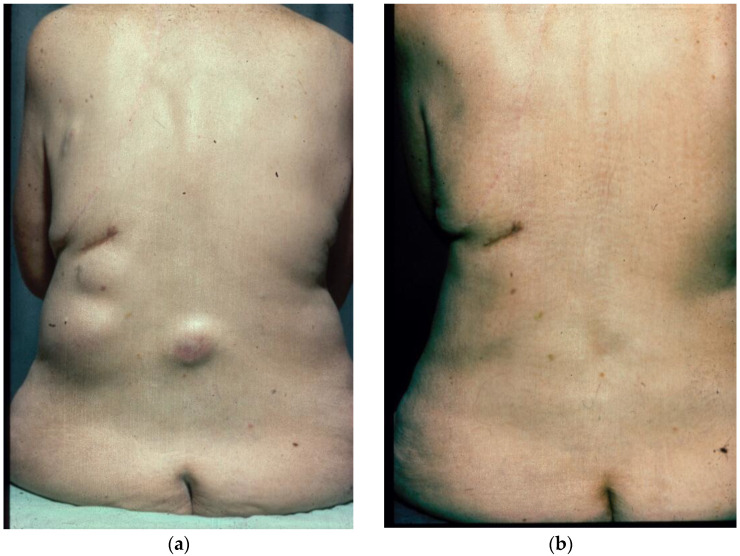
Multiple subcutaneous masses from metastatic melanoma. Before, (**a**) left. And after six courses of chemotherapy with vindesine, (**b**) right.

**Figure 2:**
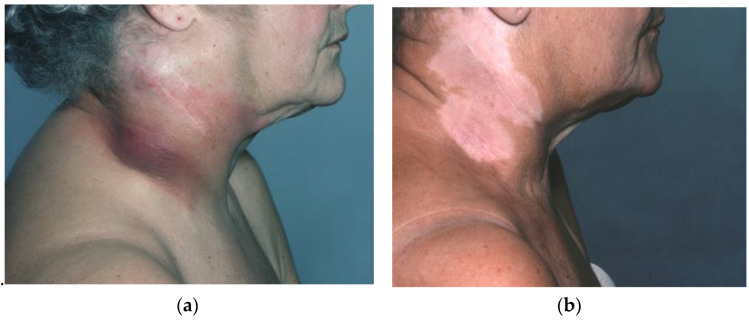
Metastatic melanoma. Progression after radiotherapy, left, (**a**) Response to combination chemotherapy with cisplatin–vinblastine–vindesine, right, (**b**).

**Figure 3:**
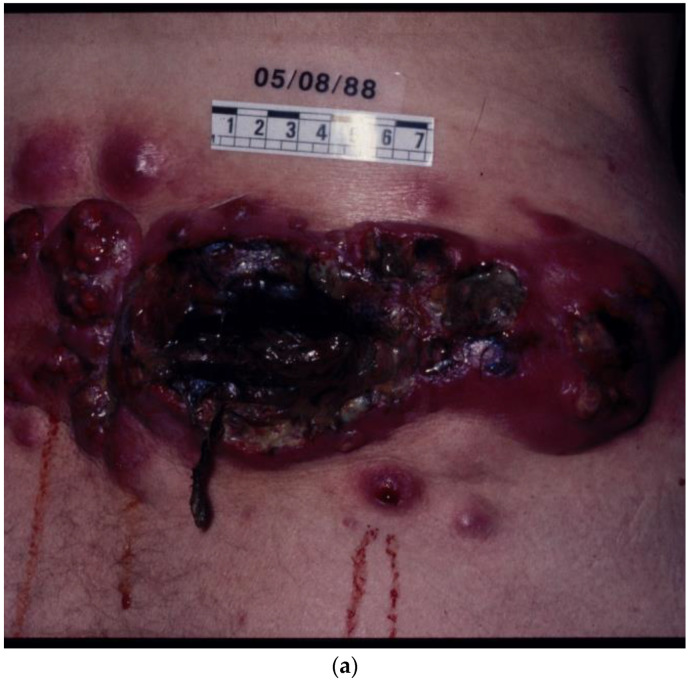

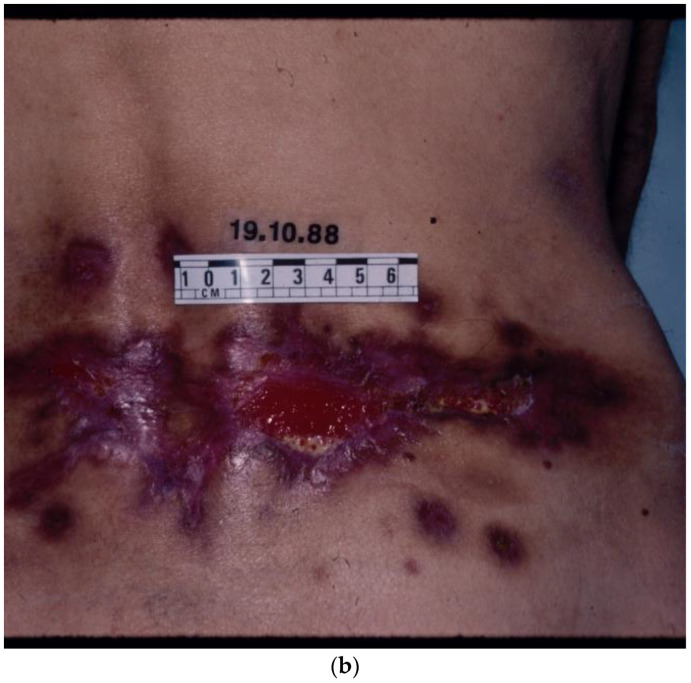
(**a**,**b**) What the ancients would have called a “therioma”. Before and after chemotherapy.

**Figure 4:**
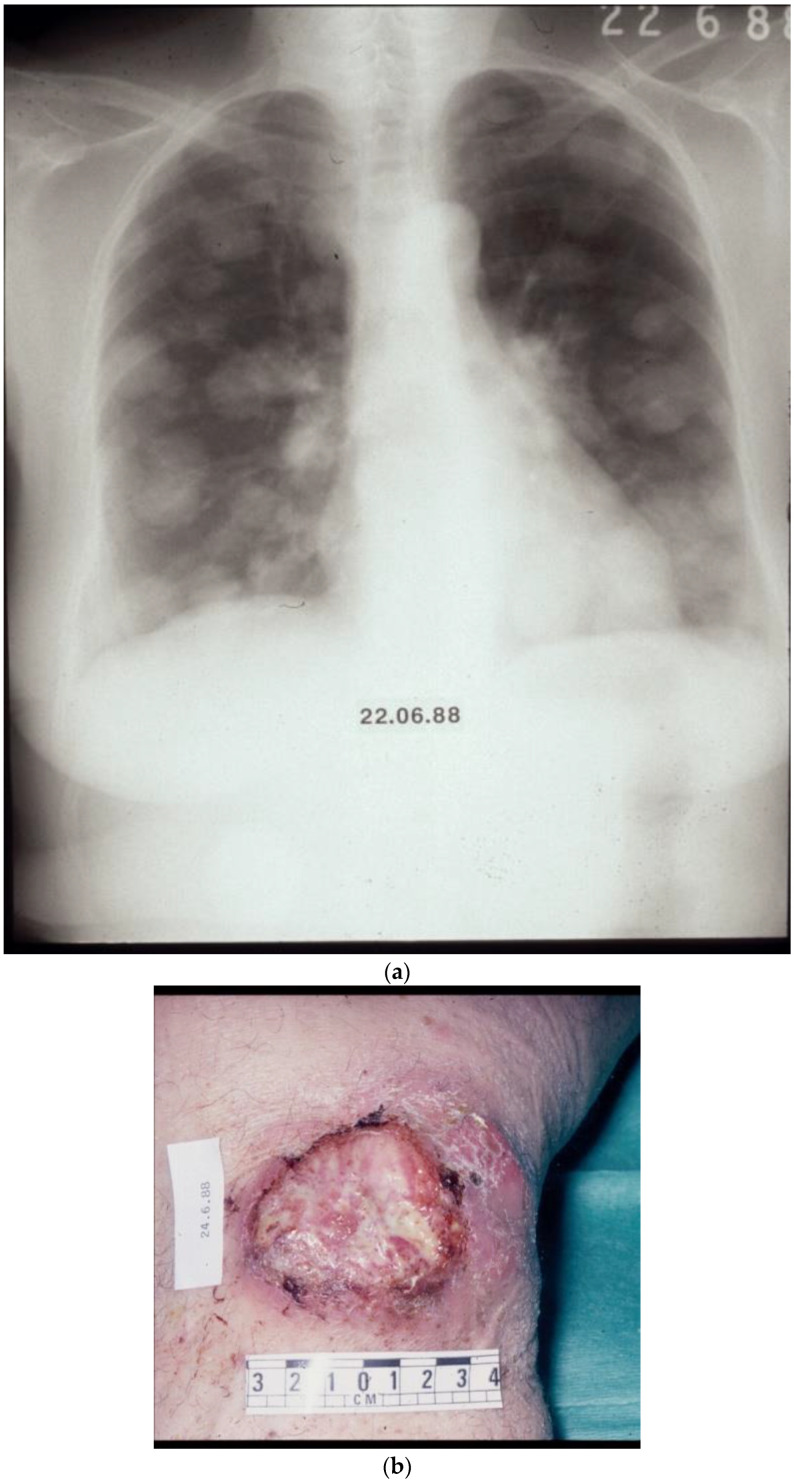

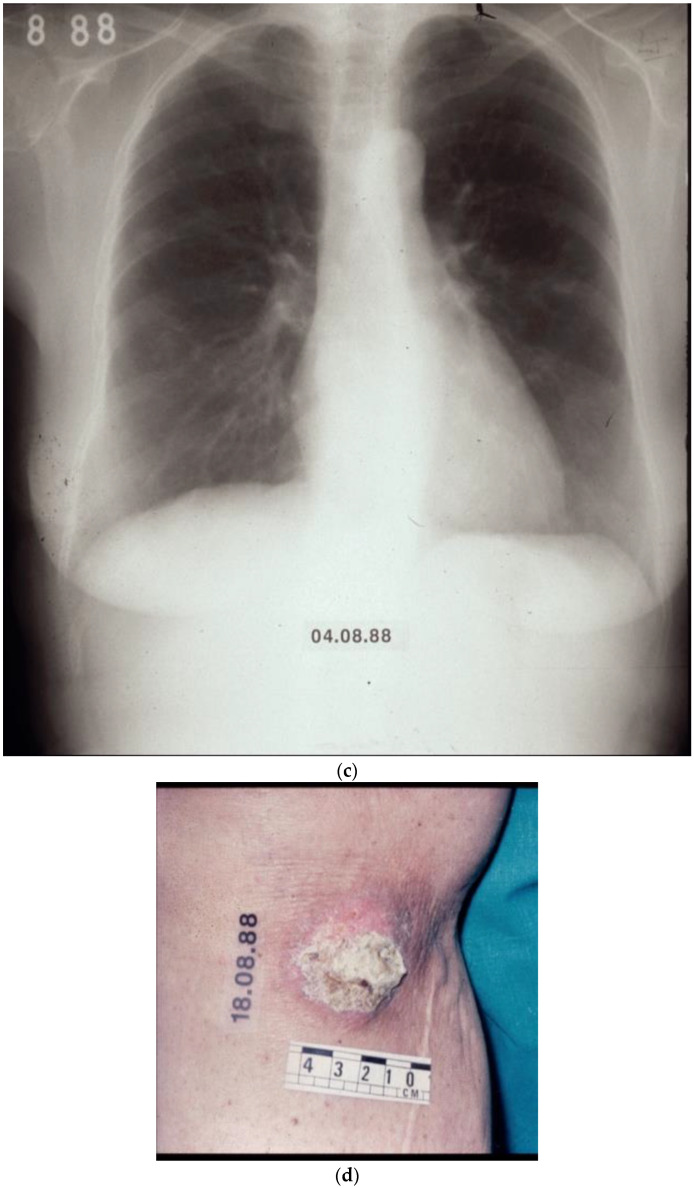
(**a**,**b**) Before treatment. (**c**,**d**) After treatment.

Examples of what, in the past, could be argued constituted “palliative” chemotherapy for a tumour broadly considered notoriously refractory to treatment are illustrated in Figure 1, Figure 2, Figure 3 and Figure 4.

Figure 1a,b—The dramatic resolution of large subcutaneous masses in a patient with disseminated melanoma before and after 6 courses of treatment with the vinca alkaloid drug, vindesine, in the 1970s.

Figure 2a,b—The target lesion in this patient progressed after radiotherapy.

Both lesions shown responded to combination chemotherapy with cisplatin–vinblastine–vindesine. Chemotherapy caused complete alopecia, but hair regrew upon the cessation of treatment.

Figure 3a,b—The case of a patient with numerous visceral and cutaneous lesions, some ulcerating with odorous discharge, intolerable to the patient and the carers. This patient was referred from a palliative care unit to the Westminster Hospital in London, UK. Figure 3a illustrates a selected lesion before the commencement of chemotherapy with the DJV3 combination, (dacarbazine, vincristine, vindesine, vinblastine, carboplatin) and Figure 3b, after treatment, with significant palliation of the target lesion shown and the complete resolution of other masses. (Image reproduced in colour from Retsas, S. et al. [23].)

Figure 4a,b—This patient had disseminated melanoma with multiple pulmonary lesions and an ulcerating mass on the right knee, the site of the primary lesion. Treated in the late 1980s with combination chemotherapy with the DJV3 combination (see above, Figure 3a,b). Resolution with treatment of pulmonary lesions and healing of the soft tissue mass overlying the right knee. (Image reproduced and supplemented in colour from Retsas, S. et al. [23].)

All pictures displayed in this communication, originating from the archives of the Medical Illustration Department of Westminster Hospital, in London, UK, were obtained with the patients’ full and written consent.

**Figure 5:**
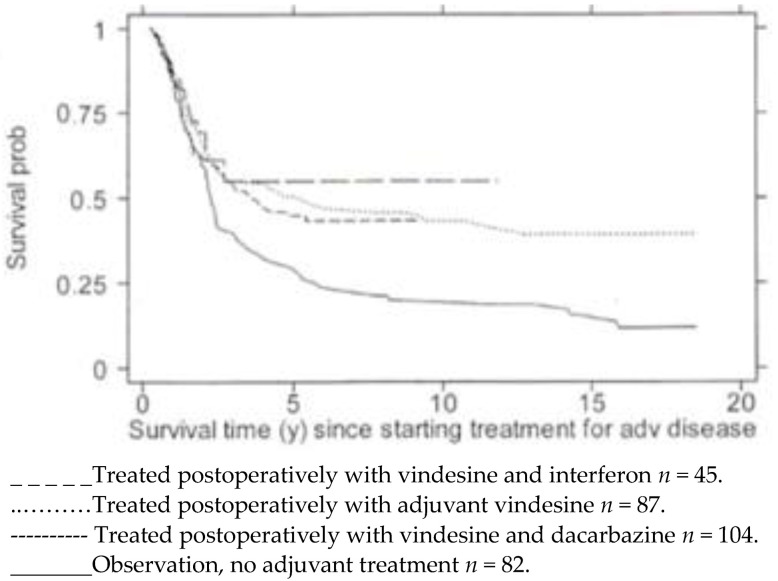
The data, obtained from the melanoma unit at Charing Cross Hospital, Chelsea and Westminster and Westminster Hospitals in London, were independently analysed by Professor Patrick Royston (MRC Clinical Trials Unit, 18 June 2001).

Figure 5 shows the observed survival in years, from a single institution, of 318 patients with metastatic melanoma from the time of diagnosis of the *clinical* and pathological involvement of regional lymph nodes, AJCC stage III disease. Survival is calculated in years, from the onset of systemic treatment with three sequential treatment protocols, following the complete resection of lymph nodes in the involved basin (*n* = 236) or no adjuvant treatment (*n* = 82). Treatment was not randomly allocated. Some patients who did not receive adjuvant treatment for stage III disease may have subsequently been treated upon widespread dissemination with treatments, as described in Figure 1 and Figure 2.

These results were presented and discussed at the 4th International Conference on The Adjuvant Therapy of Malignant Melanoma, held at the Royal College of Physicians of London on 15–16 March 2002 [24]. This analysis followed an earlier report that included the first 169 patients of this cohort [25]. The full MRC report of this analysis is available on request.

Despite the plethora of anti-cancer agents, at the end of the last century, only a small number of disseminated solid tumours were curable with the available systemic treatments: choriocarcinoma, lymphoma, and germ cell tumours, as mentioned above. As we approach the conclusion of the first quarter of the 21st century, endowed with new insights into the neoplastic process at the molecular and genomic level, and with indisputable improvements in the response rates and the duration of such responses, the number of curable cancers, so far, has hardly increased.

Although pre-operative systemic treatment—often referred to in the literature as *neoadjuvant*—was attempted with limited success in selected tumours in the 20th century [26], the higher response rates and more durable responses with the newer treatments of the 21st century make neoadjuvant systemic treatments an increasingly appealing option for some solid neoplasms [27,28,29].

But the 20th century also witnessed the emergence of targeted therapy. By recruiting an ancient drug, arsenic trioxide, into the treatment of one of the most lethal haematological malignancies, acute promyelocytic leukaemia became curable for the majority of sufferers [30,31,32,33]!

The evolving scene of systemic treatments for cancer in the last century was addressed in an interesting dissertation on the origins of the cancer cell by an eminent British oncologist [34].

The newer sequencing technologies at the dawn of the 21st century have opened a new era of therapeutic intervention, broadly, as well as at the level of personal genomes.

The development and refinement of liquid biopsies taking advantage of body fluids such as blood, saliva, or urine is generating new opportunities for non-invasive diagnosis and follow up, especially of tumours with high risk for relapse [35].

Beginning with iodine-131 for the diagnosis and treatment of thyroid cancer, the more recent application of radiopharmaceuticals—so-called *theranostics* or *theragnostics*—is contributing to the management of some neuroendocrine tumours and prostate cancer [36]. Theranostics refers to a material that serves the combined diagnosis, treatment, and follow up of a disease.

Gene therapy for cancer treatment encompasses different approaches such as immunotherapy, oncolysis mediated by viruses, and gene transfer [37,38].

Cell-based therapies using chimeric antigen receptor T cells (CAR-T) are the latest approaches in the genomic era, with some notable responses in haematologic malignancies and refractory B and plasma cell lymphomata, but so far without successful application in solid tumours. This novel therapeutic approach is not without considerable toxicity, which, in addition to other methodological issues, limits its application in common solid tumours [39,40,41,42]. Concern has also been raised about the risk, albeit rare, of the development of secondary neoplasms following CAR-T cell therapy [39,40,41,42].

The recent report of the restoration of hearing in a child with congenital deafness is an example of intervention with gene transfer into the personal genome [43]. We may anticipate parallel applications in oncology in the future, especially in cases of hereditary cancer [44,45].

New ethical issues are now emerging regarding hereditary cancers [46] affecting not only the patients but also close family members and relatives; their impact is being addressed and debated [45]. Time will decide about potential risks and unanticipated long-term side-effects from the intrusion into the genome and similar interventions.

As well as spectacular advances already seen in the treatment of cancer, puzzles in the biology of the disease await answers that will, hopefully, be provided by further advances in the study of the human genome in the near future, both in health and disease.

In our time, we wonder about the behaviour of metastasis, a biological process, characterised by some investigators as *highly inefficient*, since only 0.01% of circulating tumour cells eventually succeed in forming secondary tumours [47,48].

We are still puzzled by the tendency of ocular melanoma to metastasize to the liver in 95% of cases, some 20 years, or very much longer, after ablation of the primary focus or enucleation of the affected eye, while the same neoplasm rarely metastasizes to the anatomically adjacent brain [49,50]. Is the metastatic behaviour of this particular cancer the result of hematogenous spread, or is it a heterotopic, after decades, revival of the neoplasm, related to some hitherto unknown tissue “*sympathy*” between the choroid and the hepar?

Perhaps newer advances in the biology of cancer from the study of the human genome will, one day, decode this asymmetric relationship between the eye and the liver.

However, advances in cancer treatment do not in themselves suffice. More essential is their universal availability and efforts to decrease inequalities in cancer therapeutics, a guiding and noble objective for every oncologist [51,52].
